# Effects of nicotinamide on follicular development and the quality of oocytes

**DOI:** 10.1186/s12958-022-00938-x

**Published:** 2022-04-21

**Authors:** Ziyu Guo, Jihong Yang, Guangping Yang, Ting Feng, Xinyue Zhang, Yao Chen, Ruizhi Feng, Yun Qian

**Affiliations:** 1 Reproductive Center of Second Affiliated Hospital of Nanjing Medical University, Nanjing, 210011 China; 2 State Key Laboratory of Reproductive Medicine, Nanjing Medical University, Nanjing, 211166 China; 3 The Second Affiliated Hospital of Nanjing Medical University, Nanjing, 210011 China

**Keywords:** Female infertility, Nicotinamide, Follicular fluid, Follicle size, Oocyte quality, Oxidative stress

## Abstract

**Background:**

Nicotinamide (NAM) is an important antioxidant, which is closely related to female fertility, but its role has not been clearly elucidated. The purpose of the present study was to investigate the effects of NAM on follicular development at different stages and the quality of oocytes.

**Methods:**

The concentration of NAM in follicular fluid (FF) of 236 women undergoing in vitro fertilization (IVF) was ascertained by enzyme-linked immunosorbent assay (ELISA), and the correlation between NAM and clinical indexes was analyzed. During the in vitro maturation (IVM) of mice cumulus-oocyte complexes (COCs), different concentrations of NAM were added to check the maturation rate and fertilization rate. The reactive oxygen species (ROS) levels in the oocytes treated with different hydrogen peroxide (H_2_O_2_) and NAM were assessed. Immunofluorescence staining was performed to measure the proportion of abnormal spindles.

**Results:**

The level of NAM in large follicles was significantly higher than that in small follicles. In mature FF, the NAM concentration was positively correlated with the rates of oocyte maturation and fertilization. Five mM NAM treatment during IVM increased maturation rate and fertilization rate in the oxidative stress model, and significantly reduced the increase of ROS levels induced by H_2_O_2_ in mice oocytes.

**Conclusions:**

Higher levels of NAM in FF are associated with larger follicle development. The supplement of 5 mM NAM during IVM may improve mice oocyte quality, reducing damage caused by oxidative stress.

**Supplementary Information:**

The online version contains supplementary material available at 10.1186/s12958-022-00938-x.

## Introduction

Follicular fluid (FF) is an important constituent of the oocyte microenvironment, providing the required nutritional support for the normal growth and development of follicles and oocytes, and maintaining the normal conduction of various signaling pathways in the cells [1, 2]. FF is composed of the secretions of granulosa and theca cells combined with plasma exudates. The determination of metabolites in FF is often regarded as a reliable method for the evaluation of exposure to factors affecting reproductive outcomes [3, 4]. A recent study on oxidative lipid metabolomics of FF pointed out that fifteen oxidative lipid metabolites closely related to the arachidonic acid metabolic pathway in patients with decreased ovarian reserve were significantly lower than those in the normal ovarian reserve group, indicating that dysfunction of oxidative lipid metabolism was closely related to ovarian reserve function [5]. Yang et al. analyzed the metabolic characteristics of FF from differentially-sized follicles using liquid chromatography-tandem mass spectrometry and found that various metabolites (such as dehydroepiandrosterone) were significantly correlated with follicular development and size, oocyte maturation and the quality of embryos [6].

Nicotinamide (NAM) is a kind of B group semi-essential vitamin which can be synthesized de novo by the metabolism of tryptophan in the liver. NAM is the precursor of the important coenzyme nicotinamide adenine dinucleotide (NAD+) and nicotinamide adenine dinucleotide phosphate in the cellular energy transport chain [7], which regulates cell metabolism, redox, mitochondrial homeostasis, and energy production [8]. NAM is easily absorbed into the skin, blood, and intestines, becoming widely distributed throughout the body. Its main metabolites include N1-methylnicotinamide and N1-methyl-4-pyridone-3-carboxamide which are primarily excreted through the urinary tract [9]. Supplementation with NAM is considered to be associated with resolution of a variety of systemic diseases, including skin diseases [10], mental system degenerative diseases [11], and inflammatory diseases [12, 13]. In recent years, NAM has been found to play a variety of important beneficial roles in female reproduction. Nejabati et al. demonstrated that in letrozole-induced polycystic ovary syndrome (PCOS) in rats, compared with the control group, supplementation with NAM or the NAM metabolite N1-methylnicotinamide caused a reversal of abnormal estrus, a reduction in gene expression levels of the rate-limiting enzyme CYP17A1 that regulates androgen production and serum testosterone levels through activation of AMPK-mediated pathways [14]. It has been reported that 3 months after the oral administration of a novel dietary supplement containing NAM in patients with endometriosis, the symptoms of pelvic pain, as measured using a visual analog scale (VAS), reduced significantly and the serum levels of prostaglandin E2 and CA125 had declined significantly [15].

Oxidative stress caused by changes in components of the ovarian microenvironment affects the quality and function of oocytes and increases oocyte aging and infertility [16, 17]. Oxidative stress also affects the structure and function of proteins, lipids, and DNA, disrupting cellular activity, even resulting in cell death [18]. On the contrary, antioxidants are able to reduce the levels of reactive oxygen species (ROS) and prevent germ cell apoptosis mediated by oxidative stress. Cao et al. reported that in a mouse model of aging, quercetin was found to eliminate oxidative stress in oocytes via the sirtuin3 dependent superoxide dismutase 2 deacetylation pathway, reduce apoptosis and autophagy, and promote oocyte maturation and blastocyst formation in vitro [19]. Pang et al. found that exposure to paraquat significantly increased ROS levels and the rate of early apoptosis in bovine oocytes, reduced glutathione levels, and prevented maturation of the oocyte nucleus and cytoplasm. Addition of the antioxidant melatonin effectively reversed the reduction of oocyte quality caused by paraquat [20]. Therefore, the balance of ROS and antioxidants within the ovaries has a dramatic impact on the quality of oocytes.

A number of studies have shown that the mechanism of action of NAM is principally through antioxidant and anti-inflammatory pathways, including maintaining mitochondrial membrane potential and preventing oxidative damage and inflammation [21–24]. However, the role of NAM in FF has not yet been reported and it still remains unclear whether NAM enhances oocyte quality by the regulation of oxidative stress. The present study was aimed to explore the effects of NAM on follicle development, oocyte maturation, and the outcome of pregnancy. Additionally, we further confirmed the effect and possible mechanism of NAM at the cellular level in vitro. The results of the study will inform new strategies and form the theoretical basis for improved assisted reproductive diagnosis and treatment.

## Materials and methods

### Participants

A total of 236 women who had received IVF treatment in the Reproductive Medicine Center of the Second Affiliated Hospital of Nanjing Medical University from April 2019 to November 2020 were included in the study. The subjects were women in good overall health who were infertile due to tubal factors or unknown causes. Their male spouses had provided normal sperm for in vitro fertilization (Table S1 and Table S2). The exclusion criteria included endometriosis, premature ovarian failure, chromosomal abnormalities, hypertension, diabetes and other chronic diseases. The study was approved by the ethics committee of the Second Affiliated Hospital of Nanjing Medical University. Each subject provided written informed consent.

### FF samples

FF samples were collected as described previously [6]. The 34–36 h after intramuscular injection of human chorionic gonadotropin, fluid in the follicles was extracted while guided by transvaginal ultrasound. In each subject, one small follicle (mean diameter: 8–13 mm, FF volume: 0.3–1.0 ml) was aspirated using an unused needle, after which the total FF volume was placed in a sterile 15 ml test tube. After the initial ovarian puncture, the puncture needle was flushed and air was repeatedly drawn in until all liquid in the puncture needle had been removed. Initial aspiration of large follicles was then performed (mean diameter: 17–22 mm; FF volume: 2.5–5 ml) from the second ovary. The volume of FF collected was recorded and correlated with the diameter of the corresponding follicle. If the volume of FF did not match the size of the corresponding follicle, the sample was not included in the study. Large follicles were considered mature follicles [25], and the collection of mature FF ensured that only fluid in the large follicles was withdrawn. FF samples were centrifuged at 10000×g for 10 min, after which the supernatant was collected and stored at − 80 °C until subsequently analyzed.

### Determination of NAM concentration

The concentration of NAM in the FF was measured using an enzyme-linked immunosorbent assay (ELISA) kit (SenBeiJia Biological Technology Co., Ltd., Nanjing, China), in accordance with the manufacturer’s instructions. The absorbance of individual wells at 480 nm was recorded and quantified against a standard curve. Coefficients of variation within and between batches were 7.5 and 9.5%, respectively.

### Animal ethics and experimentation design

The experimental animal research was approved by the ethics committee of Nanjing Medical University. Unless otherwise stated, all chemicals used in this study were purchased from Sigma Aldrich Chemical Company (St. Louis, Missouri, USA).

Female ICR mice aged 3–4 weeks and males aged 9–10 weeks were separately placed in indoor ventilated cages with a 12 h light/12 h dark cycle at a constant temperature of 21–22 °C. The animals were provided sufficient drinking water and feed. There were three main experimental schemes: Experiment 1: To study the effective concentration of NAM in the oocytes during in vitro maturation (IVM). Cumulus expansion, mother cell maturation rate, and fertilization rate were evaluated after co-culture with immature oocytes in IVM medium with the addition of 0.01, 0.1, 1, 5 or 10 mM NAM for 16–18 h [26–29], in com-parison to a control. Experiment 2: To establish a murine oxidative stress model. Oocytes were pretreated with different concentrations of hydrogen peroxide (H_2_O_2_; 0, 20, 50, or 100 μM) for 1 h to evaluate the maturation rate and fertilization rate of the oocytes. Experiment 3: To study whether NAM was able to enhance the quality of oocytes when subjected to oxidative stress. Based on the results of the previous two experiments, IVM was conducted in culture medium supplemented with 0 mM or 5 mM NAM after pretreatment of the oocytes for 1 h with 100 μM H_2_O_2_. The same experimental conditions described above were studied, with the levels of ROS in the oocytes, and the ratio of abnormal spindle analyzed.

### Oocyte collection and in vitro maturation

To obtain oocytes that were fully developed to GV stage, the mice were injected intraperitoneally with 5 IU PMSG [30]. After 46–48 h, the mice were euthanized and the ovaries completely stripped. The antral follicles were repeatedly punctured with a 1 ml syringe, and the immature GV oocytes in the form of cumulus-oocyte complexes (COCs) were released into α-MEM medium supplemented with 3-isobutyl-1-methylxanthine to maintain the GV stage, and only oocytes with an intact cumulus cell layer were recovered. To achieve IVM, the collected COCs were first transferred to 2 ml α-MEM supplemented with 20% fetal bovine serum (FBS), 50 U/ml penicillin, and 50 μg/ml streptomycin. The oocytes were eluted from 3-isobutyl-1-methylxanthine by suction, then placed in IVM medium (5% FBS, 3 ng/ml EGF, 50 mIU/ml rFSH, 0.25 mmol/L sodium pyruvate, 0.5% penicillin, 0.5% streptomycin in α-MEM culture medium; Aibei Biotechnology co., Ltd., Nanjing, China) containing different concentrations of NAM (0, 0.01, 0.1, 1, 5 or 10 mM in experiment 1; 0 or 5 mM in experiment 3). The oocytes were cultured at 37 °C in 5% CO2 for 16–18 h. All media was equilibrated overnight at 37 °C and 5% CO2 prior to use. The maturity of the oocytes was scored following IVM. GV oocytes were identified by the presence of a nuclear membrane and nucleolus, while those with a first polar body represented MII oocytes.

### In vitro fertilization

Fertilization in vitro was performed as described previously [31]. Briefly, male mice were euthanized by cervical dislocation prior to the end of the in vitro maturation process. A syringe was used under a stereomicroscope to release sperm from the epididymal tail and vas deferens into a droplet culture dish containing 450 μl human tubal fluid (HTF, Aibei Biotechnology co., Ltd., Nanjing, China), which was then incubated at 37 °C in a 5% CO2 incubator for 30 min-1 h to allow the sperm to capacitate. COCs were transferred to a fertilization petri dish containing 245 μl HTF and approximately 5 μl of sperm. After 6–7 h, the embryos were washed three times in a washing dish to remove excess sperm. The fertilization rate was confirmed and recorded by morphological evaluation of pronucleus formation. All media were equilibrated overnight at 37 °C and 5% CO2 prior to use.

### Induction and rescue of oxidative stress

COCs were treated for 1 h with α-MEM containing different concentrations of H_2_O_2_ (0, 20, 50, or 100 μM) to induce oxidative stress, in accordance with previously published methods [32–34]. The oocytes were then transferred to IVM medium containing NAM (0 or 5 mM) for 16 h. The COCs were exfoliated from the surrounding granulosa cells by gently pipetting in 0.1% hyaluronidase, after which, mature oocytes were collected to additionally measure the level of ROS and perform immunofluorescence staining.

### Measurement of ROS levels

ROS levels in the oocytes were assessed in accordance with the manufacturer’s procedure [35]. MII oocytes subjected to different experimental conditions were incubated in α-MEM supplemented with 10 μM 2′,7′-dichlorodihydrofluorescein diacetate (Beyotime Institute of Biotechnology) at 37 °C for 30 min, then washed with PBS at least three times. Images of the oocytes were acquired using a fluorescence microscope using the same settings. Fluorescence intensity was calculated using ImageJ software.

### Immunofluorescence staining

Immunofluorescence staining was performed, as described previously [36]. Oocytes without granulosa cells were fixed in 2% paraformaldehyde for 20 min at room temperature. The oocytes were permeabilized in phosphate buffered saline (PBS) containing 0.5% Triton X-100 for 20 min, then transferred to a blocking solution containing 3% BSA for 2 h. The oocytes were incubated with primary monoclonal antibody (1:500; anti-α-tubulin) at 4 °C in the dark overnight. The oocytes were then washed three times with washing solution (PBS + 0.5% Triton X-100 + 0.5% Tween-20) then incubated with secondary antibody (1:1000; rabbit anti-mouse) at room temperature for 1 h. The oocytes were again washed after which the DNA was stained with Hoechst 33342 (Beyotime Institute of Biotechnology) for 15 min. Finally, the stained oocytes were fixed on slides using anti-quenching sealing tablets, sealed with coverslips, and stored in the dark. Representative images were captured using a laser scanning confocal microscope (Carl Zeiss Micro Imaging GmbH, Jena, Germany).

### Statistical analysis

The experimental data are expressed as mean ± the standard error of the mean (SEM). SPSS 23.0, GraphPad Prism 8.0, and Image J software were used for statistical analysis. A two-tailed paired t-test and one-way ANOVA were used for comparison between groups, while groups were correlated using Spearman correlation analysis. All experiments were repeated at least three times, and at least 20 oocytes were examined for each group. The differences between groups were considered significant at *P* < 0.05.

## Results

### Variation in NAM concentration in FF at different developmental stages

**Fig. 1:**
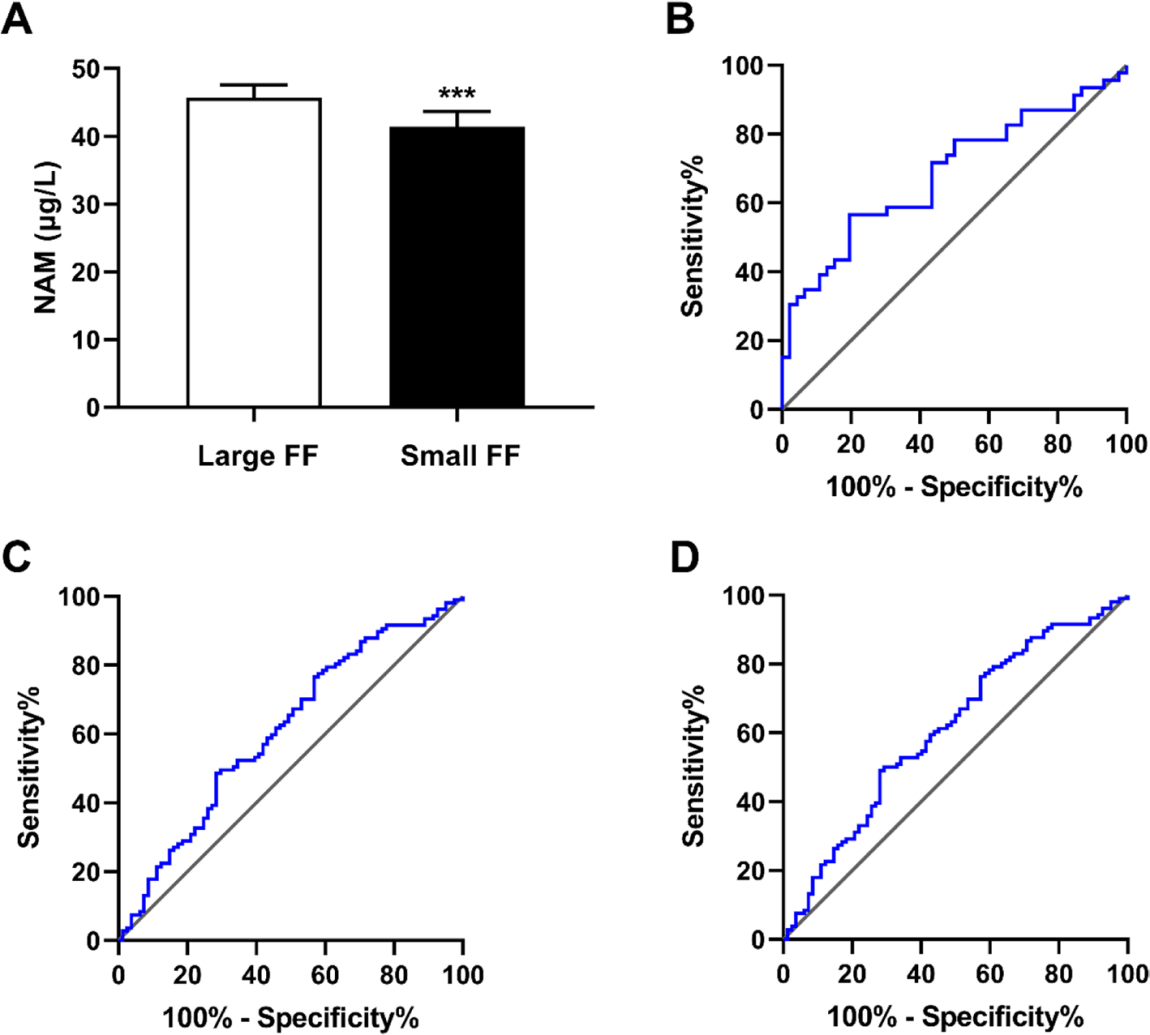
**a** NAM concentration in the follicular fluid at different stages of development. **b** Follicle size: The area under the ROC curve for NAM was 0.685, *p* = 0.0023. **c** Oocyte maturation rate: The area under the ROC curve for NAM was 0.610, *p* = 0.0099. **d** Normal fertilization rate: The area under the ROC curve for NAM was 0.611, *p* = 0.0093

A total of 46 patients were included in this experiment. The NAM concentration in large FF (*n* = 46) and small FF (*n* = 46) from the same female (*n* = 46) was measured. The results indicated that NAM concentration in large FF (45.72 ± 1.85 μg/L) was higher than that in the corresponding small FF (41.40 ± 2.29 μg/L), a statistically significant difference (*P* < 0.001, Fig. 1a).

Each pair of small FF and large FF samples were obtained from the same subject. The mean age of the women was 30.02 ± 0.52 years, with a mean body mass index (BMI) of 21.74 ± 0.42 kg/m^2^ (Table 1). In accordance with previous research methods [6], the correlation between the ratio of NAM in large FF to those in small FF (large follicles represented the control group and small follicles the experimental group) was selected as the clinical indicator for correlation analysis. The different clinical indicators displayed different correlation characteristics, with correlation coefficients and their corresponding *P* values shown in Table 1. The data demonstrate that the ratio of NAM in large FF to small FF was positively correlated with the rate of fertilization (r = 0.335, *P* = 0.023).

**Table 1:** Spearman correlation coefficients between NAM and clinical epidemiological information in large/small FF

Parameters	Values	r	*P*
Age (years)	30.02 ± 0.52	0.092	0.542
BMI (kg/m^2^)	21.74 ± 0.42	0.007	0.966
AFC (n)	16.87 ± 0.97	− 0.150	0.321
Basal LH (mIU/ml)	4.85 ± 0.47	− 0.009	0.955
Basal FSH (mIU/ml)	7.90 ± 0.44	0.152	0.320
AMH (ng/ml)	3.66 ± 0.42	0.043	0.779
Basal E2 (pg/ml)	48.49 ± 3.06	0.052	0.733
Gn duration (days)	10.22 ± 0.23	0.291	0.050
Gn dose (IU)	2375 ± 64.72	0.230	0.125
E2 on HCG day (pmol/L)	4228.61 ± 240.12	0.012	0.937
Progesterone on HCG day (nmol/L)	1.12 ± 0.11	0.177	0.240
LH on HCG day (IU/L)	0.97 ± 0.09	0.012	0.938
Oocytes retrieved (n)	13.87 ± 054	0.043	0.776
Mature oocytes (n)	10.46 ± 0.63	0.108	0.474
Oocyte maturation rate (%)	77.58 ± 2.41	0.187	0.213
Fertilization rate (%)	73.60 ± 2.35	0.335	0.023*
Cleavage rate (%)	99.69 ± 1.23	0.065	0.666
High-quality embryo rate (%)	31.70 ± 3.29	−0.251	0.092

*Values are significantly different between groups (*P* < 0.05)

*NAM* nicotinamide, *FF* follicular fluid, *BMI* body mass index, *AMH* anti-Müllerian hormone, *AFC* antral follicle count; *GnRH* gonadotrophin-releasing hormone, *HCG* human chorionic gonadotrophin; oocyte maturation rate = number of oocytes at MII stage/total number of oocytes retrieved; fertilization rate = number of fertilized embryos/total number of oocytes retrieved

### Correlation analysis between NAM concentration in mature follicles and IVF cycle characteristics

In order to study the relationship between follicular fluid NAM and oocyte quality and pregnancy outcome, samples of mature FF were collected from 190 women undergoing IVF, and the concentration of NAM was determined. The relevant clinical epidemiological information was collected for analysis. To clarify the direct relationship between NAM in FF and the important clinical indicators in the oocyte retrieval cycle, the correlation between NAM concentration and clinical indicators was analyzed. The results demonstrate that NAM concentration in mature FF was positively correlated with oocyte maturation rate (r = 0.173, *P* < 0.05) and fertilization rate (r = 0.165, *P* < 0.05), the difference of which was statistically significant (Table 2). This was consistent with the results of the first phase of the study of NAM in the FF at different stages of development, indicating that increased NAM concentration in the FF may indicate the good quality of oocytes. However, NAM levels were not related to high-quality embryo rate and clinical pregnancy rate.

**Table 2:** Correlation analysis between FF NAM and IVF cycle characteristics

Parameters	Values	r	*P*
NAM (μg/L)	51.52 ± 2.23		
Age (years)	32.16 ± 0.40	0.028	0.698
BMI (kg/m^2^)	22.54 ± 0.24	− 0.077	0.295
AFC (n)	15.29 ± 0.52	− 0.037	0.613
Basal LH (mIU/ml)	4.64 ± 0.22	− 0.052	0.475
Basal FSH (mIU/ml)	9.13 ± 0.37	− 0.021	0.776
AMH (ng/ml)	3.24 ± 0.21	− 0.012	0.873
Basal E2 (pg/ml)	48.36 ± 3.27	0.064	0.385
Gn duration (days)	10.75 ± 0.22	− 0.056	0.445
Gn dose (IU)	2358.82 ± 58.14	−0.059	0.417
E2 on HCG day (pmol/L)	3963.26 ± 271.00	0.082	0.264
Progesterone on HCG day (nmol/L)	1.04 ± 0.06	0.083	0.259
LH on HCG day (IU/L)	1.92 ± 0.20	0.042	0.570
FSH on HCG day (IU/L)	15.54 ± 0.32	0.038	0.608
Oocytes retrieved (n)	11.01 ± 0.62	0.065	0.372
Mature oocytes (n)	8.19 ± 0.49	0.121	0.095
Oocyte maturation rate (%)	74.59 ± 1.75	0.173	0.018*
Fertilization rate (%)	74.30 ± 1.75	0.165	0.024*
Cleavage rate (%)	95.44 ± 1.33	0.013	0.861
High-quality embryo rate (%)	49.75 ± 2.31	−0.024	0.744
Clinical pregnancy rate (%)	49.18 ± 3.44	0.060	0.429

*Values are significantly different between groups (*P* < 0.05)

To further explore the concentration of NAM that affected important clinical indicators of IVF in patients, the patients were grouped according to the tertiles of NAM concentration and the differences in clinical epidemiological data between the three groups compared (Table 3). The level of NAM was positively associated with that of Basal E2 (*P* = 0.001). Furthermore, the oocyte maturation and fertilization rate increased with increasing NAM concentration. As displayed in Table 3, the percentage of oocyte maturation in the lowest and highest NAM concentration groups was 67.90 ± 3.32% and 78.56 ± 2.57% (*P* < 0.05), respectively, while the percentage oocyte fertilization at both ends of the third percentile of NAM concentration was 67.84 ± 3.33% and 78.28 ± 2.57% (*P* < 0.05). Therefore, we speculate that NAM plays a beneficial role in enhancing oocyte maturation and fertilization capability.

**Table 3:** Clinical characteristics of patients by NAM concentration tertiles

Parameters	Group1	Group2	Group3	*P*
	*n* = 63	*n* = 64	*n* = 63	
NAM (μg/L)				
Median	33.61	43.88	59.38	
Range	8.57–38.78	38.8–49.29	49.39–251.15	
Age (years)	32.29 ± 0.74	32.52 ± 0.75	31.67 ± 0.59	0.674
BMI (kg/m^2^)	22.35 ± 0.41	22.86 ± 0.39	22.41 ± 0.46	0.649
AFC (n)	15.32 ± 0.85	15.84 ± 1.05	14.71 ± 0.79	0.680
Basal LH (mIU/ml)	4.76 ± 0.32	4.68 ± 0.43	4.46 ± 0.36	0.844
Basal FSH (mIU/ml)	10.07 ± 0.78	8.23 ± 0.53	9.12 ± 0.55	0.120
AMH (ng/ml)	3.11 ± 0.33	3.53 ± 0.42	3.07 ± 0.32	0.610
Basal E2 (pg/ml)	37.37 ± 2.40	65.29 ± 8.40	42.05 ± 3.42	0.001*
Gn duration (days)	10.94 ± 0.42	10.75 ± 0.37	10.57 ± 0.34	0.793
Gn dose (IU)	2386.91 ± 102.02	2350.78 ± 100.21	2338.89 ± 101.41	0.941
E2 on HCG day (pmol/L)	3666.62 ± 393.25	4027.55 ± 582.21	4198.32 ± 407.98	0.719
Progesterone on HCG day (nmol/L)	1.05 ± 0.10	1.05 ± 0.14	1.02 ± 0.07	0.975
LH on HCG day (IU/L)	1.95 ± 0.34	1.81 ± 0.34	2.00 ± 0.38	0.923
FSH on HCG day (IU/L)	16.10 ± 0.68	15.09 ± 0.54	15.44 ± 0.42	0.425
Oocytes retrieved (n)	10.94 ± 1.16	10.33 ± 1.05	11.78 ± 1.00	0.633
Mature oocytes (n)	7.46 ± 0.85	8.33 ± 0.91	8.79 ± 0.76	0.527
Oocyte maturation rate (%)	67.90 ± 3.32	77.37 ± 2.99	78.56 ± 2.57	0.023*
Fertilization rate (%)	67.84 ± 3.33	76.84 ± 2.99	78.28 ± 2.57	0.030*
Cleavage rate (%)	92.82 ± 3.09	95.93 ± 2.28	97.61 ± 0.98	0.328
High-quality embryo rate (%)	49.06 ± 4.17	51.86 ± 4.27	48.31 ± 3.58	0.805
Clinical pregnancy rate (%)	47.84 ± 6.12	46.33 ± 6.03	53.19 ± 5.80	0.688

*Values are significantly different between groups (*P* < 0.05)

### ROC curve of NAM and follicular size, oocyte maturation rate, and normal fertilization rate

A receiver operating characteristic (ROC) curve was used to predict the value of NAM for enhanced follicular development size, oocyte maturation rate, and normal fertilization rate. After analyzing the ROC curve, it was found that the areas under the curve (AUC) for follicular developmental size (AUC = 0.685, *P* = 0.0023), oocyte maturation rate (AUC = 0.610, *P* = 0.0099), and normal fertilization rate (AUC = 0.611, *P* = 0.0093) were significantly greater than the reference line (0.5). For follicle size, the best sensitivity and specificity for the ROC curve were 80.4 and 56.5% for a NAM concentration cut-off point for FF of 39.75 μg/L (Fig. 1b). A cut-off value of 46.62 μg/L for NAM concentration in FF represented a sensitivity of 48.6% and specificity of 71.6% for the prediction of oocyte maturation rate (Fig. 1c). The sensitivity and specificity of NAM concentration in predicting the fertilization rate of oocytes at a cut-off value of 46.62 μg/L was 49.1 and 72.0% (Fig. 1d), respectively.

### Effects of NAM supplementation during IVM on cumulus expansion, oocyte maturation, and IVF

**Fig. 2:**
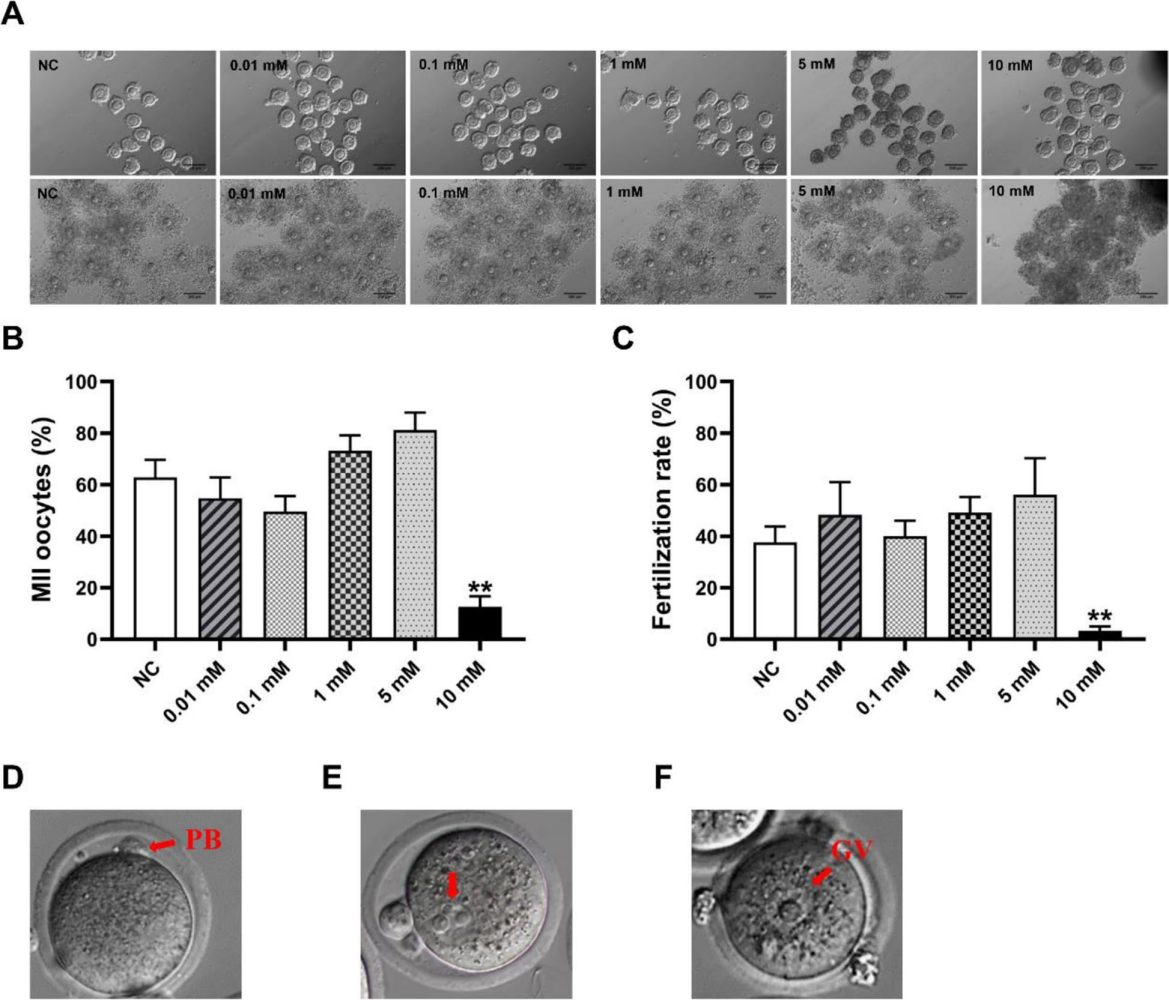
Effects of different concentrations of NAM on oocyte maturation and fertilization. The effect of NAM on cumulus expansion was observed using a light microscope (**a**). Percentages of oocytes matured (**b**) and fertilized (**c**) in vitro following treatment with NAM. **d**, **f** Representative images of mature and immature oocytes. Pronucleus formation in fertilized oocytes (**e**). PB: Polar body; GV: germinal vesicle. Values are significantly different between groups (*P < 0.05, **P < 0.01). Scale bar = 200 μm

Since NAM was found to be positively correlated with oocyte maturation and fertilization in human, in vitro experiments were used to further confirm and explore the role of NAM on reproduction. Different concentrations of NAM (0, 0.01, 0.1, 1, 5, or 10 mM) were added to the IVM medium of mouse oocytes, and the expansion of cumulus cells assessed after 16–18 h (Fig. 2a). Subsequently, the maturation and fertilization rate of the oocytes following NAM treatment were measured. Compared with the control group (62.82% ± 6.81%), NAM treatment enhanced the oocyte maturation rate (Fig. 2b), especially at 5 mM (81.22% ± 6.77%), although not significantly. The rate of fertilization of the oocytes similarly increased after treatment with NAM (Fig. 2c). Therefore, 5 mM NAM was selected as the concentration for rescue in follow-up experiments. However, we found that an excessive NAM concentration inhibited the maturation and fertilization of oocytes. The formation of MII oocytes was significantly reduced after treatment with 10 mM NAM (12.63 ± 4.09% vs 62.82% ± 6.81% for control, *P* = 0.009), while the fertilization rate also decreased significantly (3.29% ± 1.73% vs 37.60% ± 6.18% for control, *P* = 0.007).

### H_2_O_2_ exposure affects oocyte maturation and fertilization

**Fig. 3:**
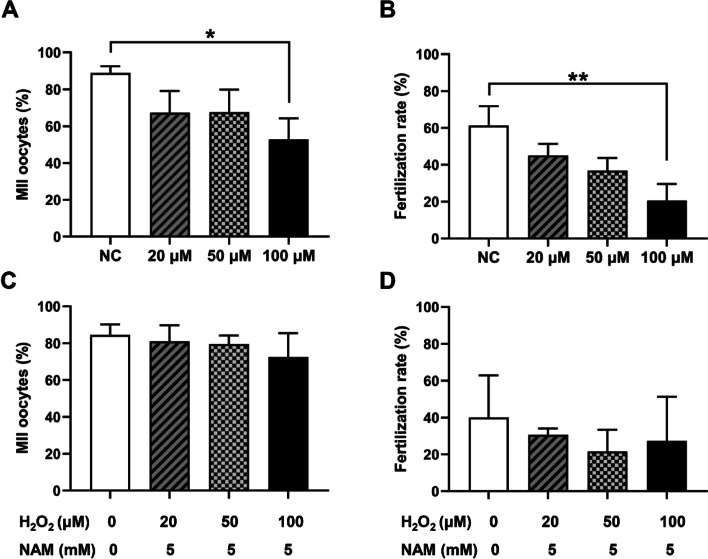
Effects of different concentrations of H_2_O_2_ on maturation and fertilization. **a** Maturation rate of oocytes treated with different concentrations of H_2_O_2_ in vitro. **b** Fertilization rate of oocytes treated with different concentrations of H_2_O_2_ in vitro. The effects of NAM on the maturation rate of H_2_O_2_-exposed oocytes (**c**) and the fertilization rate (**d**). Values were significantly different between groups (*P < 0.05, **P < 0.01)

To further confirm our hypothesis that NAM may play a beneficial role in the reproductive system through its antioxidant properties, H_2_O_2_ was used to construct an in vitro oxidative stress injury model. The oocytes were pretreated with 0, 20, 50, or 100 μM H_2_O_2_ for 1 h prior to IVM, and the subsequent maturation and fertilization rate of the oocytes measured. As displayed in Fig. 3a, the maturation rate of the oocytes after H_2_O_2_ treatment (67.25% ± 11.89, 67.65% ± 12.19, and 52.80% ± 11.47% for 20, 50, and 100 μM, respectively), was lower than that of the control group (88.89% ± 3.64%), which reached a significant level at 100 μM (*P* = 0.04). As H_2_O_2_ concentration increased, the oocyte fertilization rate decreased in a dose-dependent manner (Fig. 3b), which declined significantly in the 100 μM H_2_O_2_ treatment group (20.60% ± 8.98% vs 61.33% ± 10.41% for control, *P* = 0.008). It is possible that H_2_O_2_ treatment impaired the maturation and fertilization of oocytes in vitro. H_2_O_2_ treatment at 100 μM met our subsequent experimental requirements.

### NAM rescues the maturation and fertilization of oocytes exposed to H_2_O_2_

To explore the protective effects of NAM on H_2_O_2_-induced oocyte inhibition, 5 mM NAM was used to co-treat the oocytes in additional experiments. As expected, NAM effectively prevented a decrease in oocyte maturation and fertilization rate induced by H_2_O_2_. Although the oocyte maturation rate and fertilization rate of each experimental group were still lower than those of the control group, the difference was not statistically significant (*P* > 0.05). Combined with results of H_2_O_2_-induced injury alone (Fig. 3a, b), NAM supplementation in vitro was shown to resist the damage by H_2_O_2_ to oocytes to a certain extent, partially restoring the maturation and fertilization capability of H_2_O_2_-exposed oocytes (Fig. 3c, d).

### NAM attenuates H_2_O_2_-induced oxidative stress and spindle abnormality in oocytes

**Fig. 4:**
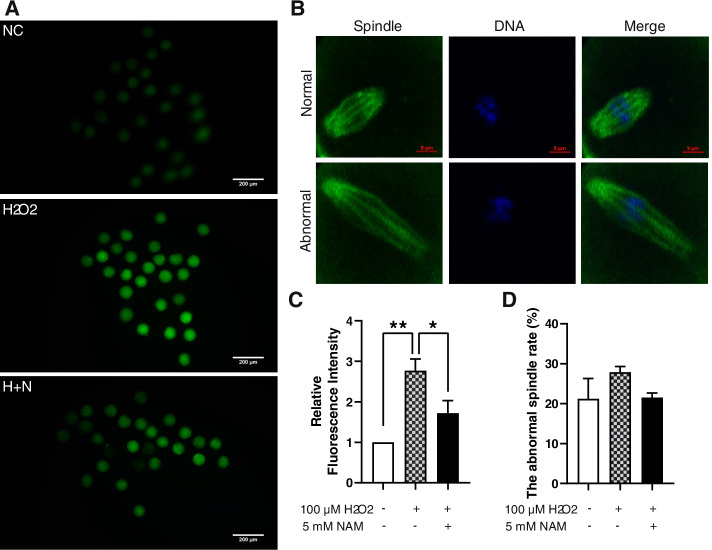
NAM treatment impacted ROS levels and spindle abnormality in oocytes damaged by oxidative stress during IVM. Fluorescence staining for ROS (**a**) and relative fluorescence intensity (**c**) of MII oocytes in each group (Scale bar = 200 μm). **b** Typical images of spindle morphology and chromosome arrangements in different groups. **d** Spindle deformity rate in each group (Scale bar = 5 μm). The asterisk indicates a statistical significance (*P* < 0.05)

To determine the efficacy of NAM in improving H_2_O_2_-induced oxidative stress, the level of ROS in MII oocytes was measured using ROS detection kit. As shown in Fig. 4c, compared with the control group, 100 μM H_2_O_2_ treatment significantly increased the level of ROS in oocytes (*P* < 0.01) (The marking in Fig. 4 is always correct.), while the addition of 5 mM NAM significantly reduced the high level of oxidative stress induced by 100 μM H_2_O_2_ (*P* < 0.05). This suggests that exogenous NAM supplementation lowered the extent of oxidative stress in oocytes when exposed to stress, reducing the damage to oocyte quality.

In addition, to elucidate the efficacy of NAM in reducing H_2_O_2_-induced oocyte spindle damage, immunofluorescence staining was used to investigate spindle morphology in the oocytes. Although not a significant effect, the rate of abnormalities in spindle formation in oocytes exposed to H_2_O_2_ increased, while additional NAM supplementation reduced the rate of abnormalities in H_2_O_2_-exposed oocytes (Fig. 4b, d), indicating that NAM may prevent spindle abnormality in oocytes exposed to H_2_O_2_ to a certain extent.

## Discussion

In the present study, the concentrations of NAM in FF ranged from 8.57 μg/L to 251.15 μg/L, with a mean value of 51.52 μg/L. The distribution of NAM in large and small follicles varied, with the concentrations of NAM in large FF significantly higher than that in small FF. Moreover, the NAM levels in mature FF were positively correlated with oocyte maturation and fertilization rate. These observations were then validated in an oxidative stress model, the results suggesting that NAM supplementation enhanced oocyte maturation and fertilization, down-regulating the degree of oxidative stress in mice oocytes. Previous studies have shown that the NAM concentration in mammalian tissues is very low, at approximately 11–400 μM [37]. However, the NAM concentration in the human female reproductive system has not so far been measured. To the best of our knowledge, the present study quantified NAM in human FF for the first time and explored the relationship between NAM and clinical parameters related to assisted reproduction.

Changes in the composition of FF metabolites can affect follicular development and oocyte quality. A previous study on FF metabolomics of bovine preovulatory follicles demonstrated that 18 differential metabolites involved in glucose and amino acid metabolism were significantly positively correlated with follicle diameter and affected the developmental capability of oocytes [38]. Nakanishi et al. found that cortisol accumulation in FF resulted in the apoptosis of granulosa cells and cumulus cells, causing follicular atresia [39]. In addition, supraphysiological doses of vitamin D3 treatment have been reported to promote follicular development by significantly increasing the number of large follicles and newly formed and degenerated corpus luteum in the ovaries of normal mice [40]. Consequently, we chose FF as the research sample in our study, trying to find potential biomarkers related to follicular development and oocyte quality.

Research on Metabolomics of women with poor ovarian response showed that the metabolic pathways of niacin and NAM in serum which seem to be related to ovarian reserve may be a potential strategy to evaluate ovarian function in women of childbearing age [41]. In the present study, levels of NAM in FF were significantly correlated with follicular development after induction of ovulation in IVF women, with higher concentrations of NAM tending to result in the development of larger follicles. Oocyte maturation and quality are critical to female fertility, where regulatory factors, such as some genes [42], proteins [43] and metabolites [44], have always been the focus of research in the field of assisted reproduction. Although a number of previous studies have pointed out that NAM can inhibit oocyte maturation and embryonic development [26, 45], increasing numbers of studies have confirmed that NAM at an appropriate concentration enhances oocyte quality and even the outcomes of pregnancy. El Sheikh et al. suggested that during bovine oocyte IVM, 0.1 mM NAM supplementation enhanced cumulus cell expansion, increased the percentage of MII oocytes, significantly reduced ROS, and improved subsequent embryo development by activating the sirtuin1/threonine-specific protein kinase B (AKT) signaling pathway, although high concentrations (> 10 mM) of NAM had the opposite effect [46]. It was found that 10 mM NAM treatment in the IVF medium of bovine-fertilized embryos for 3 h reduced ROS in embryonic cells and significantly increased sirtuin1 expression and the blastocyst formation rate, indicating that NAM enhanced the developmental competence of bovine embryos in vitro via antioxidant activity [47]. However, a study showed that 20 mM NAM caused ROS accumulation and mitochondrial dysfunction in bovine oocytes, impairing oocyte maturation and embryonic developmental potential [48] Furthermore, in a review, investigators have pointed out that 5 mM NAM has a positive effect on the viability and replication of cells, but NAM exceeding 20 mM causes cell apoptosis [49]. The results of our study suggested that 5 mM may be an appropriate concentration for NAM supplementation in vitro, which improved oocyte maturation and fertilization in mice.

Oxidative stress caused by the accumulation of ROS in cells is among the principal factors that affect oocyte quality, leading to oocyte aging and a decline in fertility [50–52]. Lappas et al. found that NAM enhanced the gene expression of antioxidant enzymes, such as superoxide dismutase, and reduced lipopolysaccharide-mediated inflammatory cytokine production and oxidative stress by activating FoxO3 in the human placenta [53]. In addition, in rat fatty hepatocytes, NAM was found to reduce oxidative stress and lipid accumulation in hepatocytes via a reduction in the overexpression of glucose-6-phosphate dehydrogenase, thereby slowing hepatic steatosis [54]. Min et al. found that NAM supplementation of aged *Caenorhabditis elegans* caused a decrease in ROS accumulation, increasing the mitochondrial function of oocytes and protecting the growth and movement capability of the subsequent offspring [55]. Similarly, the present study demonstrated that 5 mM NAM supplementation in vitro protected oocyte quality by reducing the level of ROS in oocytes in the oxidative stress model.

NAM is converted to NAD + through nucleotide rescue pathway [56], which is considered to be the main source of NAD + in mammals [57]. Previous studies have proposed that NAM plays a beneficial role mainly by increasing the levels of NAD + [58]. The increase of NAD + level can reduce the production of mitochondrial ROS by regulating the activity of NAD + consuming enzyme sirtuin1 [59]. As NAD+ and sirtuin1 activity levels decline with age [8], targeted increase of NAD+ levels has become a potential therapeutic approach to ameliorate aging-related diseases and prolong human lifespan. Thus, we speculate that NAM may protect follicular development and oocyte quality by increasing endogenous NAD + levels. However, the dynamic process of mutual transformation between NAM and NAD + is extremely complicated. The mechanism of how NAM supplementation in vitro regulates NAD+ and its related enzymatic pathways in oocytes remains to be further studied, which will be the focus of our follow-up research.

Previous studies have pointed out that although the recommended dosage of NAM is considerably higher than that of other vitamins [49], NAM supplementation is safe and well-tolerated [60, 61]. However, in this study, the clinical sample size here was nevertheless too small, and only an approximate active concentration of NAM has been confirmed in an animal model. Further large sample experiments are still required to confirm the safety and effectiveness of NAM.

## Conclusions

In conclusion, our study showed that higher levels of NAM in FF are associated with larger follicle development. The NAM levels in mature FF were positively correlated with oocyte maturation rate and fertilization rate. The supplement of 5 mM NAM during IVM may improve oocyte maturation and fertilization, and reduce oocyte quality damage caused by oxidative stress in mice. The present research has provided a preliminary theoretical basis for NAM as a potential biomarker and effective nutritional support for clinical infertility patients.

## Supplementary Information

**Additional file 1: Table S1.** Clinical characteristics of male whose spouses provided large FF and small FF.

**Additional file 2: Table S2.** Clinical characteristics of male whose spouses provided mature FF.

## Data Availability

The data supporting the results of this study can be obtained from the corresponding authors upon reasonable request.

## Abbreviations

NAM: nicotinamide
AFC: Antral follicle count
AMH: Anti-Müllerian Hormone
AUC: Area under the curve
BMI: Body mass index
E2: Estradiol
FF: Follicular fluid
LH: Luteinizing hormone
FSH: Follicle-stimulating hormone
Gn: Gonadotropin
GnRH-a: Gonadotropin-releasing hormone agonist
GSH: Glutathione
HCG: Human chorionic gonadotropin
IVF: In vitro fertilization
IVM: In vitro maturation
