# Supplementary material for: Effects of nicotinamide on follicular development and the quality of oocytes
Source: Reprod Biol Endocrinol. 2022 Apr 21;20:70. doi: 10.1186/s12958-022-00938-x (PMC9022236; doi:10.1186/s12958-022-00938-x)
Supplement: Supplementary file 1 — Additional file 1: Table S1. Clinical characteristics of male whose spouses provided large FF and small FF. [file 12958_2022_938_MOESM1_ESM.docx]

**Table S1** Clinical characteristics of male whose spouses provided large FF and small FF

| Parameters | Values |
| --- | --- |
| n | 46 |
| Age (years) | 31.98 ± 0.72 |
| BMI (kg/m²) | 23.87 ± 0.37 |
| Semen volume (ml) | 3.73 ± 0.23 |
| Sperm Density (* 10^6^/ml) | 127.09 ± 14.80 |
| Sperm motility (%) | 81.93 ± 1.04 |
| Sperm deformity (%) | 83.23 ± 0.44 |
| Sperm fragmentation rate (%) | 14.69 ± 1.94 |

FF = follicular fluid. Data are presented as mean ± SEM.
