# Supplementary material for: Effects of nicotinamide on follicular development and the quality of oocytes
Source: Reprod Biol Endocrinol. 2022 Apr 21;20:70. doi: 10.1186/s12958-022-00938-x (PMC9022236; doi:10.1186/s12958-022-00938-x)
Supplement: Supplementary file 2 — Additional file 2: Table S2. Clinical characteristics of male whose spouses provided mature FF. [file 12958_2022_938_MOESM2_ESM.docx]

**Table S2** Clinical characteristics of male whose spouses provided mature FF

| Parameters | Values |
| --- | --- |
| n | 190 |
| Age (years) | 33.47 ± 0.45 |
| BMI (kg/m²) | 25.05 ± 0.24 |
| Semen volume (ml) | 3.43 ± 0.12 |
| Sperm Density (* 10^6^/ml) | 100.86 ± 5.37 |
| Sperm motility (%) | 80.07 ± 0.61 |
| Sperm deformity (%) | 81.41 ± 0.29 |
| Sperm fragmentation rate (%) | 14.35 ± 1.04 |

Data are presented as mean ± SEM.
